# *Neurog1* and *Neurog2* coordinately regulate development of the olfactory system

**DOI:** 10.1186/1749-8104-7-28

**Published:** 2012-08-20

**Authors:** Tarek Shaker, Daniel Dennis, Deborah M Kurrasch, Carol Schuurmans

**Affiliations:** 1 Hotchkiss Brain Institute, Alberta Children’s Hospital Research Institute, University of Calgary, Health Sciences Centre, 3330 Hospital Drive NW, Calgary, Alberta, T2N 4N1, Canada

**Keywords:** Olfactory bulb, Olfactory epithelium, Proneural genes, Neuronal fate specification, Neuronal migration, Axonal innervation

## Abstract

**Background:**

Proneural genes encode basic helix–loop–helix transcription factors that specify distinct neuronal identities in different regions of the nervous system. In the embryonic telencephalon, the proneural genes *Neurog1* and *Neurog2* specify a dorsal regional identity and glutamatergic projection neuron phenotype in the presumptive neocortex, but their roles in cell fate specification in the olfactory bulb, which is also partly derived from dorsal telencephalic progenitors, have yet to be assessed. Given that olfactory bulb development is guided by interactions with the olfactory epithelium in the periphery, where proneural genes are also expressed, we investigated the roles of *Neurog1* and *Neurog2* in the coordinated development of these two olfactory structures.

**Results:**

*Neurog1/2* are co-expressed in olfactory bulb progenitors, while only *Neurog1* is widely expressed in progenitors for olfactory sensory neurons in the olfactory epithelium. Strikingly, only a remnant of an olfactory bulb forms in *Neurog1*^−/−^*;Neurog2*^−/−^ double mutants, while this structure is smaller but distinguishable in *Neurog1*^−/−^ single mutants and morphologically normal in *Neurog2*^*−/−*^ single mutants. At the cellular level, fewer glutamatergic mitral and juxtaglomerular cells differentiate in *Neurog1*^−/−^*;Neurog2*^−/−^ double-mutant olfactory bulbs. Instead, ectopic olfactory bulb interneurons are derived from dorsal telencephalic lineages in *Neurog1*^−/−^*;Neurog2*^−/−^ double mutants and to a lesser extent in *Neurog2*^*−/−*^ single mutants. Conversely, cell fate specification is normal in *Neurog1*^*−/−*^ olfactory bulbs, but aberrant patterns of cell proliferation and neuronal migration are observed in *Neurog1*^*−/−*^ single and *Neurog1*^−/−^*;Neurog2*^−/−^ double mutants, probably contributing to their altered morphologies. Finally, in *Neurog1*^*−/−*^ and *Neurog1*^−/−^*;Neurog2*^−/−^ embryos, olfactory sensory neurons in the epithelium, which normally project to the olfactory bulb to guide its morphogenesis, fail to innervate the olfactory bulb.

**Conclusions:**

We have identified a cell autonomous role for *Neurog1/*2 in specifying the glutamatergic identity of olfactory bulb neurons. Furthermore, *Neurog1* (and not *Neurog2*) is required to guide olfactory sensory neuron innervation of the olfactory bulb, the loss of which results in defects in olfactory bulb proliferation and tissue morphogenesis. We thus conclude that *Neurog1/2* together coordinate development of the olfactory system, which depends on tissue interactions between the olfactory bulb and epithelium.

## Background

The olfactory system is the part of the central nervous system that is responsible for detecting and processing odors. In vertebrates, the olfactory system consists of three major components: the olfactory epithelium (OE), the olfactory bulb (OB), and the olfactory cortex. Odor molecules are initially detected by olfactory sensory neurons (OSNs) in the OE, which project their axons to the OB, where odor signals are refined and enhanced before being relayed to the piriform/olfactory cortex, where signal processing and odor perception occurs.

The OB is a ventroanterior protrusion of the cerebrum that serves as an intermediate processing center for olfactory signals. It is comprised of projection neurons and interneurons, each with distinct embryonic origins. Mitral and tufted cells are glutamatergic projection neurons that arise from dorsal telencephalic (that is, pallial) progenitors between embryonic day (E) 11 and E13 in mouse [1-3]. At E13.5, pallial progenitors also give rise to glutamatergic juxtaglomerular cells, which function as excitatory interneurons [1]. Later, at ~ E14.5, inhibitory OB interneurons, including periglomerular cells and granule cells, begin to differentiate in the lateral ganglionic eminences (LGEs) of the ventral telencephalon, migrating tangentially into the OB [4-6]. Smaller numbers of interneurons are also derived from the ventricular zone (VZ) of the OB [7], and from subependymal progenitors lining the lateral ventricles throughout life [8,9].

Development of the OB and OE are intimately intertwined. The OE is populated by OSNs that send pioneer axons to infiltrate the primordial OB beginning at ~ E11.5 in mouse [10,11]. Signals derived from pioneer OSNs are thought to reduce relative rates of cell proliferation in the rostral telencephalon, resulting in OB evagination and tissue morphogenesis [11], events that depend on Fgfr1 signaling [12]. There is also evidence that OSN innervation influences neuronal migration in the OB, as revealed by *Dlx5Fezf1* and *Arx* mutations, all of which display defects in OSN innervation that are accompanied by the generation of a smaller OB and aberrant interneuron migration [13-16].

The proneural genes *Neurog1* and *Neurog2* encode basic helix–loop–helix transcription factors that specify a dorsal regional identity and glutamatergic neurotransmitter phenotype in the neocortex [17-19]. Mitral, tufted and juxtaglomerular cells are labeled in *Neurog1* and *Neurog2* lineage traces, indicative of a pallial origin for these OB neurons [1,20]. While *Neurog1* mutants have been reported to develop a smaller OB [21], the underlying cellular defects have not been characterized, and the role of *Neurog2* in OB development has yet to be assessed. Moreover, while there is a partial loss of OSNs in *Neurog1*^*−/−*^ OEs [22,23], it is not known whether the remaining OSNs differentiate normally. Here we find that *Neurog1/2* are required in a redundant fashion to specify the identities of glutamatergic OB neurons, including mitral and juxtaglomerular cells. Conversely we show that only *Neurog1* is required for OB morphogenesis and to promote the differentiation of OSNs and their subsequent innervation of the OB. *Neurog1/2* thus coordinately regulate development of the olfactory system.

## Results

### *Neurog1* and *Neurog2* are co-expressed in glutamatergic lineages in the developing olfactory bulb

**Figure 1:**
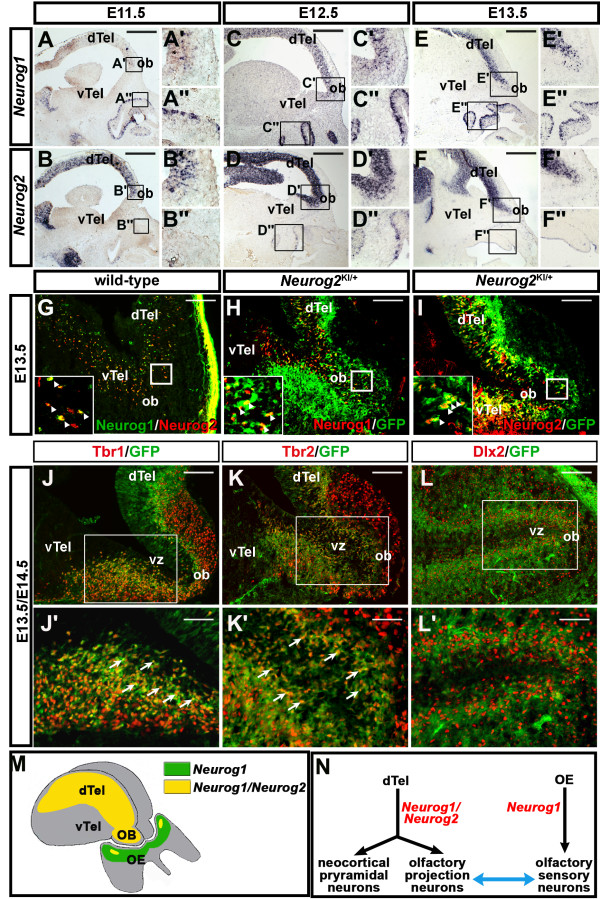
***Neurog1 *and *Neurog2 *expression in the embryonic olfactory system.** (**A**) to (**F**) Sagittal sections of embryonic day (E) 11.5, E12.5 and E13.5 embryos, showing the distribution of *Neurog1* (A-A", C-C", E-E") and *Neurog2* (B-B", D-D", F-F") transcripts. Insets in A to F are fourfold magnifications of the boxed areas in the OB (**A'** to **F'**) and OE (**A"** to **F"**). **(G) to (I)** Co-immunolabeling of E13.5 *Neurog2 *^*KI/*+^ brains with antibodies to Neurog1 and Neurog2 (**G**), Neurog1 and GFP (**H**), and Neurog2 and GFP (**I**). Insets in (**G**) to (**I**) are fourfold magnifications of the boxed areas. (**J**) to (**L**) Co-immunolabeling of the E13.5 OB with Tbr1 and GFP (**J**,**J'**) and with Tbr2 and GFP (**K**,**K'**), and labeling of the E14.5 OB with Dlx2 and GFP (**L**,**L'**). Boxed areas in (**J**) to (**L**) are magnified twofold in **J'**, **K'**, **L'** respectively. **(M)** Schematic representation of Neurog1 single-positive OE progenitors (green) and Neurog1/Neurog2 double-positive progenitors (yellow) in the OB and a small region of the OE. **(N)** Schematic illustration of the objectives of this study; to determine the roles of *Neurog1* and *Neurog2* in olfactory system development. dTel, dorsal telencephalon; OB, olfactory bulb; OE, olfactory epithelium; vTel, ventral telencephalon. Scale bars: 1 mm (**A**) to (**F**), 250 μm (**G**) to (**L**), 125 μm (**J'**) to (**L'**).

The proneural genes *Neurog1* and *Neurog2* are co-expressed in dorsal telencephalic (that is, pallial) progenitors [18,19,24], including those that give rise to glutamatergic neuronal lineages in the neocortex and OB [1,20]. To begin to assess how *Neurog1* and *Neurog2* might function together during OB development, we first compared their expression profiles at three key time points: E11.5, prior to the onset of OB differentiation; E12.5, when OB morphogenesis has initiated and mitral cell projection neurons are differentiating, and E13.5, when the first juxtaglomerular cells are born [1-3,25]. At E11.5, *Neurog1* transcripts were detected in only a few cells in the VZ of the dorsal telencephalon, including in the primordial OB at the rostral-most edge (Figure 1A-A"). In contrast, *Neurog2* was expressed throughout the E11.5 pallial VZ, including in the presumptive OB (Figure 1B-B"). By E12.5 and at E13.5, when the OB is visible as a morphological protrusion [11,26], the number of neocortical and OB VZ cells expressing *Neurog1* steadily increased (Figure 1C-C",E-E"), while *Neurog2* expression remained widespread throughout the neocortical and OB VZs (Figure 1D-D",F-F"). Notably, at all stages analyzed, *Neurog1* was also widely expressed throughout the basal OE (Figure 1A-A",C-C",E-E"), as previously documented [22], whereas *Neurog2* expression was limited to a small, ventromedial OE domain (shown at E12.5; Figure 1D"**)**.

Immunostaining at E13.5 confirmed that Neurog1 and Neurog2 proteins were indeed co-expressed in pallial progenitors, including in the presumptive neocortex, as previously demonstrated [24], and in the developing OB (Figure 1G). Recent long-term and short-term fate-mapping studies have indicated that *Neurog1*[20] and *Neurog2*[1] are expressed in all glutamatergic neuronal lineages in the OB, including mitral and tufted cell projection neurons and juxtaglomerular cells in the glomerular layer (GL). To determine to what extent Neurog1 and Neurog2 were expressed in the same or different OB lineages, we used a *Neurog2GFP* knock-in (KI) allele (*Neurog2*^KI^) to perform short-term GFP-lineage tracing of *Neurog2*-expressing cells and their progeny [24]. The vast majority (if not all) Neurog1-positive (Figure 1H) and Neurog2-positive (Figure 1I) VZ progenitors in the OB co-expressed GFP, suggesting that Neurog1 and Neurog2 are indeed co-expressed within the same OB lineage(s). GFP expression also persisted in *Neurog2*^+/*KI*^ OB cells migrating out of the VZ, including those cells that had stopped expressing Neurog1 and Neurog2, allowing the fate of these cells to be assessed with molecular markers (Figure 1J,K,L). GFP^+^ cells in the mantle layer of the E13.5 *Neurog2*^+/*KI*^ OB co-expressed Tbr1 (Figure 1J,J') and Tbr2 (Figure 1K,K'), markers of dorsally-derived, glutamatergic neurons [27,28], as recently reported [1]. In contrast, GFP^+^ cells did not express the ventral-specific regional marker Dlx2 in E14.5 *Neurog2*^+/*KI*^ embryos (Figure 1L,L').

These data demonstrate that Neurog1 and Neurog2 are largely co-expressed in pallial progenitors, including those that give rise to Tbr1^+^ and Tbr2^+^ glutamatergic neurons in the developing OB (Figure 1M). In contrast, only Neurog1 is expressed to a significant extent in OE lineages (Figure 1M), raising the question of how these proneural genes coordinately regulate development of the olfactory system (Figure 1N).

### OB morphogenesis and lamination are disrupted in *Neurog1*^*−/−*^ and *Neurog1/2*^−/−^ embryos

**Figure 2:**
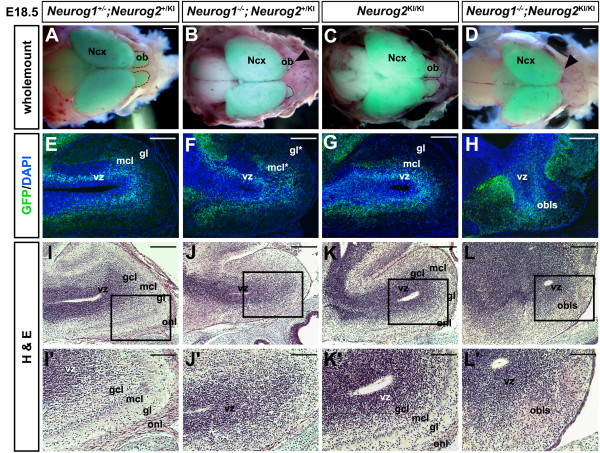
**Defects in olfactory bulb morphogenesis and lamination in *Neurog1 ***^***−/−***^**and *Neurog1 ***^***−/−***^***;Neurog2 ***^**KI/KI**^**mutant embryos.** (**A**) to (**D**) Whole-mount dorsal views of partially dissected embryonic day (E) 18.5 wild-type (**A**), *Neurog1*^−/−^ (**B**), *Neurog2 *^KI/KI^ (**C**), and *Neurog1/2 *^−/−^ (**D**) brains, all heterozygous or homozygous for a *Neurog2*^*GFPKI *^ allele. Brains were left in the cranium and imaged by merging bright-field and GFP fluorescent images. Arrowheads mark the reduction in OB size in *Neurog1*^*−/−*^ embryos (B), and apparent loss of the OB in *Neurog1/2 *^−/−^ embryos (D). (**E**) to (**H**) GFP epifluorescence (green) and nuclear DAPI staining (blue) of sagittal sections through E18.5 wild-type (**E**), *Neurog1*^−/−^ (**F**), *Neurog2*^KI/KI^ (**G**), and *Neurog1/2*^−/−^ (**H**) OBs. (**I**) to (**L**) H & E histological analysis of E18.5 wild-type (**I**,**I'**), *Neurog1*^−/−^ (**J**,**J'**), *Neurog2*^KI/KI^ (**K**,**K'**), and *Neurog1/2*^−/−^ (**L**,**L'**) OBs. (**I'**) to (**L'**) are twofold magnifications of the boxed areas in (**I**) to (**L**), respectively. GCL, granule cell layer; Gl, glomerular layer; MCL, mitral cell layer; NCX, neocortex; OB, olfactory bulb; OBLS, olfactory bulb-like structure; ONL, outer nerve layer; VZ, ventricular zone. Scale bars: 2 mm (A) to (D), 500 μm (**E**) to (**L**), 250 μm (**I'**) to (**L'**).

To determine whether *Neurog1* and *Neurog2* are required for OB development, we used a loss-of-function approach, analyzing *Neurog1*[29] and *Neurog2*^*GFP*KI^[24] single and double null mutants. In E18.5 wild-type (Figure 2A) and *Neurog2*^*KI/KI*^ mutant (Figure 2C) embryos, the OB was visible as a distinct morphological protrusion of the ventroanterior brain. In comparison, the OB was much smaller in *Neurog1*^*−/−*^ embryos (Figure 2B), and a morphologically distinct OB was not apparent in *Neurog1*^*−/−*^*;Neurog2*^*KI/KI*^ double mutants (*Neurog1/2*^*−/−*^; Figure 2D). To examine OB development at the cellular level, we first monitored GFP expression from the *Neurog2*^KI^ allele, which serves as a short-term lineage trace of mitral, tufted and juxtaglomerular lineages [1]. In E18.5 double heterozygotes and *Neurog2*^*KI/KI*^ and *Neurog1*^*−/−*^ null mutants (the latter maintained on a *Neurog2*^*KI/+*^ background), GFP-labeled cells were detected in the OB VZ and developing mitral cell layer (MCL). In *Neurog1*^−/−^ OBs, GFP^+^ cells in the glutamatergic OB lineages were disorganized and formed a less distinct MCL (Figure 2E,F,G). Strikingly, in sections through the *Neurog1/2*^*−/−*^ double-mutant forebrain, an OB-like structure (OBLS) with a central ventricle that was surrounded by GFP^+^ cells was detected in an aberrant location in the ventrolateral brain (Figure 2H). To further characterize the laminar organization of the proneural mutant OBs, E18.5 sagittal sections were stained with H & E. In H & E-stained wild-type (Figure 2I,I') and *Neurog2*^*KI/KI*^ (Figure 2K,K') mutant OBs, a distinct VZ, granule cell layer, MCL, GL and outer nerve layer (ONL) were apparent. In contrast, most of the post-mitotic neuronal layers were indistinct in the E18.5 *Neurog1*^*−/−*^ OB (Figure 2J,J') and *Neurog1/2*^*−/−*^ OBLS (Figure 2L,L'), although a VZ and granule cell layer were discernible in both mutants.

We thus conclude that *Neurog1* is required for proper growth and lamination of the OB, whereas *Neurog1* and *Neurog2* are together required for overall OB morphogenesis. We set out to identify the underlying cause(s) for the morphological and laminar defects in these proneural mutants.

### Defects in the migration of glutamatergic neurons in *Neurog1*^−/−^ OBs and migration and differentiation in *Neurog1/2*^−/−^ OBLSs

**Figure 3:**
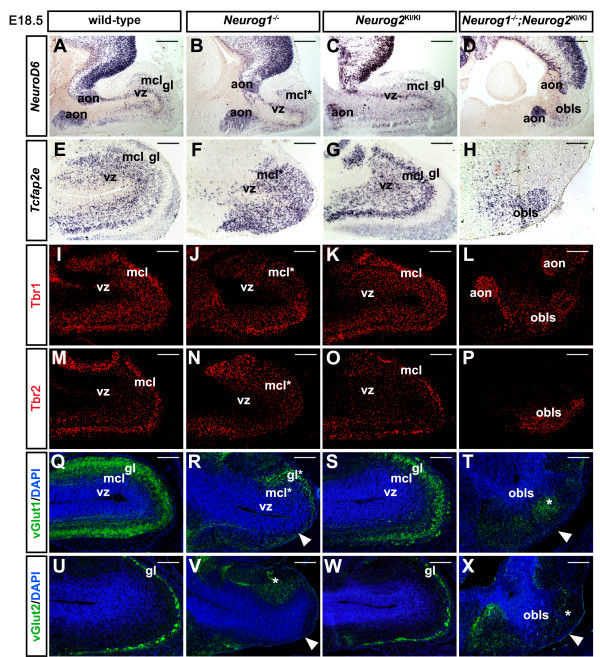
**Impaired lamination and differentiation of excitatory neurons in *Neurog1/2 ***^***−/− ***^**single and double mutant olfactory bulbs.** (**A**) to (**X**) Expression of *NeuroD6* (**A**) to (**D**), *Tcfap2e* (**E**) to (**H**), Tbr1 (**I**) to (**L**), Tbr2 (**M**) to (**P**), vGlut1 (green)/DAPI (blue) (**Q**) to (**T**) and vGlut2 (green)/DAPI (blue) (**U**) to (**X**) in embryonic day 18.5 wild-type (A,E,I,M,Q,U), *Neurog1*^*−/−*^ (B,F,J,N,R,V), *Neurog2*^KI/KI^ (C,G,K,O,S,W) and *Neurog1/2*^−/−^ (D,H,L,P,T,X) olfactory bulbs (OBs). White arrowheads in R,V,T,X mark diminished vGlut1/2 protein expression in the peripheral glomerular layer (GL). AON, anterior olfactory nucleus; MCL, mitral cell layer; OBLS, olfactory bulb-like structure; VZ, ventricular zone. Scale bars: 1 mm (**A**) to (**D**), 500 μm (**E**) to (**X**).

The disruption of lamination in E18.5 *Neurog1*^−/−^ OBs and *Neurog1/2*^*−/−*^ OBLSs suggested that the neuronal subtypes that populate these layers may not differentiate properly. To test this, we first examined glutamatergic OB lineages, which are derived from *Neurog1*-expressing and *Neurog2*-expressing pallial progenitors, including projection neurons (mitral and tufted cells) and interneurons (juxtaglomerular cells) (see above, and [1,20]). To label projection neurons in the MCL, we used a panel of dorsal telencephalic-specific markers, including *NeuroD6Tcfap2eNrp1NeuroD1Reelin*, Tbr1 and Tbr2 (Figure 3 and data not shown). Notably, *Tcfap2e* also labels OB progenitors, and is one of the few definitive markers of an OB identity as it is not also expressed in neocortical lineages [30], unlike the rest of the markers we employed. To unambiguously identify the OBLS in *Neurog1/2*^*−/−*^ double mutants, the anterior olfactory nucleus (AON), which lies between the neocortex and OB, was used as a landmark. In E18.5 *Neurog2*^KI/+^ embryos, the AON was labeled by GFP (data not shown), indicating that it is also derived from *Neurog2*-expressing pallial progenitors. In all E18.5 *Neurog1/2* single and double mutants, the AON expressed GFP (data not shown), *Neurod6* (Figure 3A,B,C,D) and Tbr1 (data not shown), indicating that AON development is not grossly perturbed by the loss of these proneural genes.

In the main OB, expression of *NeuroD6*, *Tcfap2e*, Tbr1 and Tbr2 was detected in the OB VZ and MCL in E18.5 wild-type and *Neurog2*^*KI/KI*^ null embryos (Figure 3A,C,E,G,I,K,M,O). In contrast, *NeuroD6*, *Tcfap2e*, Tbr1 and Tbr2-expressing cells were generated, but were disorganized in E18.5 *Neurog1*^−/−^ OBs, occupying ectopic positions in the outermost portion of the OB, where a mitral cell-deficient GL would normally form (Figure 3B,F,J,N). Strikingly, *NeuroD6*, *Tcfap2e*, Tbr1 and Tbr2 expression was also detected in the aberrantly localized OBLS in E18.5 *Neurog1/2*^*−/−*^ embryos, although the number of *Tcfap2e*-positive cells was markedly reduced (Figure 3D,H,L,P). *Neurog1/2* are thus required for the lamination of MCL projection neurons in the OB, and may together be required for the differentiation of these cells.

We next asked whether *Neurog1/2* were required for the differentiation of glutamatergic juxtaglomerular cells in the GL, which includes external tufted and short axon cells that are labeled by vesicular glutamate transporter 1 (vGlut1) and vGlut2 [1,31,32]. In E18.5 wild-type (Figure 3Q,U) and *Neurog2*^KI/KI^ (Figure 3S,W) OBs, vGlut1 labeled a large number of juxtaglomerular cell bodies and their projections, while vGlut2 expression was confined to the ONL in the periphery of the GL. In *Neurog1*^*−/−*^ OBs, vGlut1 and vGlut2 staining was strongly reduced in the presumptive GL, and an ectopic cluster of vGlut1/2-labeled cells aggregated in the dorsal OB (Figure 3R,V). Similarly, while scattered vGlut1/2-immunoreactive cells were detected throughout the *Neurog1/2*^*−/−*^ OBLS, a distinct GL was not evident in these embryos (Figure 3T,X).

**Figure 4:**
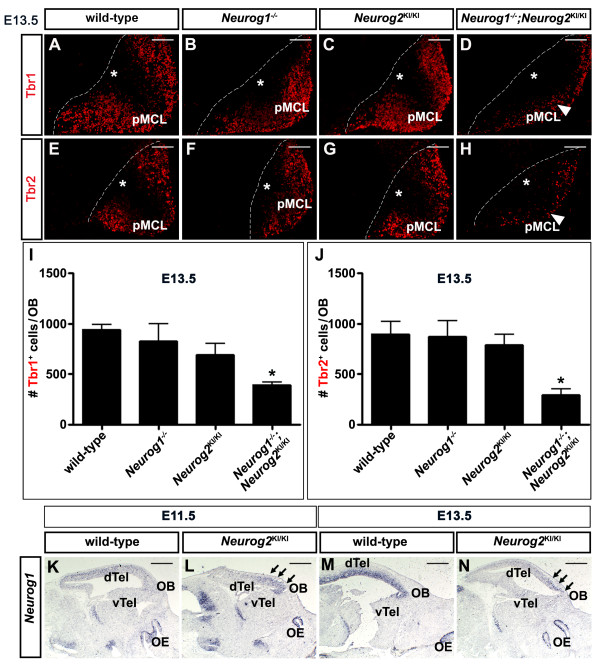
**Defects in the differentiation of glutamatergic olfactory bulb neurons in*Neurog1/2 *double mutants.** (**A**) to (**H**) Expression of Tbr1 (**A**) to (**D**) and Tbr2 (**E**) to (**H**) in the presumptive MCL of embryonic day (E) 13.5 wild-type (**A**,**E**), *Neurog1*^*−/−*^ (**B**,**F**), *Neurog2 *^*KI/KI*^ (**C**,**G**) and *Neurog1/2 *^−/−^ (**D**,**H**) OBs. White arrowheads in D,H mark the reduction in glutamatergic neurons in *Neurog1/2*^−/−^ OBs. White asterisks in (**A**) to (**H**) mark the ventricular zone. **(I), (J)** Quantitation of total numbers of Tbr1^+^ cells (I) and Tbr2^+^ cells (**J**) in E13.5 wild-type *Neurog1*^*−/−*^, *Neurog2*^KI/KI^ and *Neurog1/2*^−/−^ OBs. Asterisks denote *P* <0.05. (**K**) to (**N**) Analysis of *Neurog1* expression in E11.5 (K,L) and E13.5 (M,N) wild-type and *Neurog2*^*−/−*^ telencephalons. Arrows mark ectopic *Neurog1* expression in (**L**) and (**N**). Scale bars: 250 μm (**A**) to (**H**), 1 mm (**K**) to (**N**).

Finally, to quantitate glutamatergic neurons in the OB, we analyzed the expression of Tbr2, a pan-glutamatergic neuronal marker, and Tbr1, which labels MCL projection neurons and short axon juxtaglomerular cells in the GL (Figure 4A to J). Cell counts were performed at E13.5, when the vast majority of glutamatergic OB neurons have differentiated [1-3]. We observed a significant reduction in the number of Tbr1^+^ (wild-type and *Neurog2*^*KI/KI*^*n* = 4; *Neurog1*^*−/−*^ and *Neurog1/2*^*−/−*^*n* = 3) and Tbr2^+^ (wild-type and *Neurog2*^*KI/KI*^*n* = 4; *Neurog1*^*−/−*^ and *Neurog1/2*^*−/−*^*n* = 3) glutamatergic neurons only in the *Neurog1/2*^*−/−*^ double-mutant OBLS compared with wild-type OBs (*P* <0.05 for both Tbr1 and Tbr2 counts; Figure 4I,J).

We thus conclude that glutamatergic mitral and juxtaglomerular cells are born in normal numbers in *Neurog2*^*KI/KI*^ and *Neurog1*^−/−^ single-mutant OBs, but these cells migrate inappropriately and fail to take up their correct positions in the *Neurog1*^−/−^ MCL and GL. In contrast, fewer glutamatergic neurons are born in the *Neurog1/2*^*−/−*^ OBLS, and these cells also migrate aberrantly.

### *Neurog1* is upregulated in *Neurog2*^*−/−*^ olfactory bulbs

The lack of an apparent defect in the *Neurog2*^−/−^ OB (at least at the morphological level and in glutamatergic lineages) was surprising given that fewer glutamatergic neurons are generated in *Neurog2*^−/−^ single-mutant neocortices. We previously attributed the *Neurog2*^*−/−*^ neocortical phenotype to a downregulation of *Neurog1* expression in dorsomedial telencephalic domains, such that *Neurog2*^*−/−*^ and *Neurog1/2*^*−/−*^ embryos are equivalent (that is, both lack *Neurog1* and *Neurog2* expression) in this part of the developing neocortex [18]. We therefore asked whether *Neurog1* expression was similarly lost in the presumptive OB region of *Neurog2*^*−/−*^ embryos. Strikingly, we found that *Neurog1* was instead upregulated in the *Neurog2*^*−/−*^ rostral telencephalon (presumptive OB) at both E11.5 (Figure 4K,L) and to a lesser extent at E13.5 (Figure 4M,N). In contrast, *Neurog1* expression was reduced throughout most of the remainder of the *Neurog2*^*−/−*^ dorsal telencephalon, as previously documented [18]. These data are consistent with the idea that *Neurog1* may compensate for the loss of *Neurog2* in the developing OB.

### Relative rates of OB proliferation are elevated in *Neurog1*^*−/−*^ and *Neurog1/2*^−/−^ OBs

**Figure 5:**
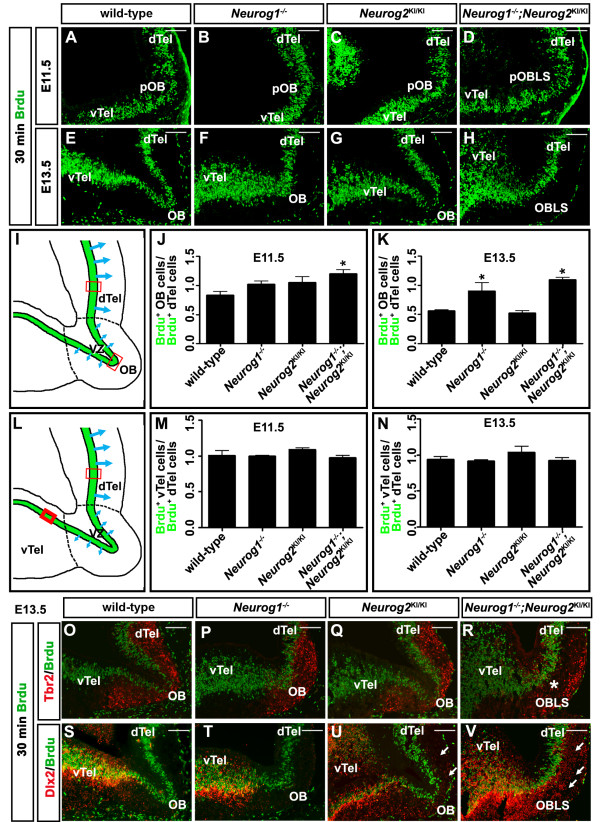
**Aberrant patterns of cell proliferation in *Neurog1/2 ***^***−/− ***^**mutants.** (**A**) to (**H**) BrdU-labeled S-phase progenitors at embryonic day (E) 11.5 **(A)** to (**D**), and E13.5 (**E**) to (**H**) in wild-type (**A**,**E**), *Neurog1*^*−/− *^ (**B**,**F**), *Neurog2 *^KI/KI^ (**C**,**G**), and *Neurog1/2*^−/−^ (**D**,**H**) OBs. (**I**) to (**K**) Illustration schematizing areas where counts of BrdU-labeled S-phase progenitors were performed (I). Quantitation of the ratio of BrdU^+^ cells in the OB compared with the dorsal telencephalon at E11.5 (**J**) and E13.5 (**K**). Asterisks denote *P* <0.05. (**L**) to (**N**) Illustration schematizing areas where counts of BrdU-labeled S-phase progenitors were performed (**L**). Quantitation of the ratio of BrdU^+^ cells in the ventral compared with the dorsal telencephalon at E11.5 (**M**) and E13.5 (**N**). (**O**) to (**V**) Co-labeling of BrdU^+^ S-phase progenitors (green) with either Tbr2 (red, O to R) or Dlx2 (red, S to V) in E13.5 wild-type (O,S), *Neurog1*^*−/−*^ (**P**,**T**), *Neurog2*^KI/KI^ (**Q**,**U**), and *Neurog1/2*^−/−^ (**R**,**V**) embryos. dTel, dorsal telencephalon; OB, olfactory bulb; pOB, presumptive olfactory bulb; pOBLS, presumptive olfactory bulb like structure; vTel, ventral telencephalon. Scale bars: 250 μm (**A**) to (**H**), (**O**) to (**V**).

Beginning at ~ E12.5, the OB is first evident as a distinct rostral protuberance of the telencephalon [11,26]. In our analysis of glutamatergic neuronal markers, we observed a shortening of the proximal–distal telencephalic axis in *Neurog1*^*−/−*^ mutants as early as E13.5, while a morphologically distinct OB was not evident in *Neurog1/2*^*−/−*^ mutants at any stage analyzed (between E12.5 and E18.5; data not shown). At these early stages, the driving force of OB morphogenesis is thought to be a reduction in proliferation at the rostral edge of the telencephalon, which results in the neocortex ballooning out while the presumptive OB is left behind [11,26]. To determine whether aberrant patterns of proliferation contributed to the morphogenetic defects observed in *Neurog1*^*−/−*^ and *Neurog1/2*^*−/−*^ OBs, dividing S-phase progenitors were labeled with a 30-minute BrdU pulse and labeled progenitors were then enumerated in fields of equal size in the presumptive neocortex (dorsal telencephalon) and OB (Figure 5A to K). The presumptive OB was identified at these early stages as the midpoint of the telencephalic continuum surrounding the lateral ventricles. Specifically, the OB is flanked by dorsal and ventral telencephalic domains, both of which have distinct morphological features, and the borders of which were precisely identified by BrdU co-labeling with Tbr2 (dorsal) or Dlx2 (ventral).

OB/dorsal telencephalic proliferation ratios were compared across all *Neurog1/2* genotypes. At E11.5, when the OB is not yet distinct, the ratio of S-phase progenitors in the OB versus dorsal telencephalon was similar in wild-type embryos (83.3 ± 6.5%, *n* = 4; Figure 5A,J), *Neurog1*^*−/−*^ embryos (102.6 ± 5.1%, *P* >0.05, *n* = 4; Figure 5B,J) and *Neurog2*^*KI/KI*^ embryos (105.0 ± 9.9%, *P* >0.05, *n* = 3; Figure 5C,J). In contrast, the ratio of BrdU-labeled progenitors in the OB versus dorsal telencephalon was 120.5 ± 6.7% in E11.5 *Neurog1/2*^*−/−*^ double mutants, 1.4-fold higher than in wild-type embryos (*P* = 0.02, *n* = 3; Figure 5D,J). OB proliferation rates are thus aberrantly high in *Neurog1/2*^*−/−*^ double mutants as early as E11.5.

We next examined proliferating pallial progenitors at E13.5, when the OB is morphologically distinct (Figure 5E,F,G,H,K). In E13.5 wild-type embryos, the ratio of BrdU-labeled VZ progenitors in the OB versus dorsal telencephalon had declined to 56.3 ± 1.6% (*n* = 5; Figure 5E,K), indicative of a reduction in relative rates of OB proliferation, consistent with previous reports [11,12,26]. In E13.5 *Neurog2*^*KI/KI*^ embryos, which develop a morphologically normal OB, a similar OB/dorsal telencephalon proliferation ratio was observed (52.8 ± 3.5%, *n* = 3; Figure 5G,K). In contrast, the ratio of proliferating progenitors in the OB versus dorsal telencephalon was aberrantly high in E13.5 *Neurog1*^*−/−*^ OBs (90.4 ± 14.0%, *P* <0.001, *n* = 3; Figure 5F,K) and *Neurog1/2 *^*−/−*^ OBLSs (109.5 ± 3.7%, *P* <0.001, *n* = 3; Figure 5H,K).

To confirm that the OB/dorsal telencephalon proliferation ratios were not altered in *Neurog1* or *Neurog2* single mutants because of a defect in the neocortex (as opposed to OB), we also compared the ratios of BrdU^+^ cells in the dorsal versus ventral telencephalon (Figure 5L,M,N). Note that neither *Neurog1* nor *Neurog2* are expressed in the ventral telencephalon, so proliferation rates should not be altered in this domain in mutants (serving as an internal control). Consistent with the lack of a defect in neocortical cell proliferation in *Neurog1/2* single and double mutants, at both E11.5 (Figure 5M) and E13.5 (Figure 5N), the ratios of BrdU-labeled ventral versus dorsal telencephalic progenitors were similar in all genotypes (*P* >0.05 for all pairwise comparisons against wild-type). We thus conclude that prospective OB progenitors fail to reduce their relative proliferation rates in *Neurog1*^*−/−*^ and *Neurog1/2*^*−/−*^ mutants, probably contributing to the observed OB morphogenesis defects.

To further characterize proliferation defects in early OB development, we examined the spatial arrangement of BrdU-labeled S-phase progenitors in the E13.5 VZ with respect to differentiating mitral cells. Early-born mitral cells migrate radially from the OB VZ, using radial glia as a scaffold, while later-born mitral cells shift to a tangential pattern of migration, coursing through the intermediate zone of the OB in close proximity to tangentially oriented axons of early-born mitral cells [10,33]. Consequently, mitral cells generated at E10 show a bias towards dorsomedial positions, while tangentially migrating cells born at E12 preferentially accumulate in ventrolateral domains. In E13.5 wild-type OBs (Figure 5O) and *Neurog2*^KI/KI^ OBs (Figure 5Q), Tbr2^+^ mitral cells had migrated throughout the mantle layer of the OB, lining the OB surface along the entire dorsal-to-ventral axis, but were less abundant in a central zone at the rostral tip. In E13.5 *Neurog1*^*−/−*^ OBs (Figure 5P), the distribution of Tbr2^+^ cells was altered, such that a Tbr2-deficient zone at the rostral tip was not observed, suggestive of early defects in cell migration. These migratory defects were more severe in E13.5 *Neurog1/2*^*−/−*^ OBLSs, in which a distinct gap was evident between the BrdU-labeled progenitor zone and the Tbr2^+^ mantle layer (Figure 5R). Migration defects are thus evident as early as E13.5 in *Neurog1*^*−/−*^ and *Neurog1/2*^*−/−*^ OBs.

### Defects in the differentiation and migration of olfactory bulb interneurons in *Neurog1*^−/−^, *Neurog2*^*KI/KI*^ and *Neurog1/2*^−/−^ mutants

In the embryonic neocortex, *Neurog1* and *Neurog2* regulate a binary fate decision, promoting a dorsal regional identity and glutamatergic neurotransmitter phenotype while repressing an alternative ventral, GABAergic neuronal identity [18,19]. We thus speculated that the reduction in glutamatergic neuronal number in the *Neurog1/2*^*−/−*^ OBLS may be due to a similar fate switch. To test this, E13.5 embryos were labeled with Dlx2, which together with *Dlx1* is required for the generation of almost all GABAergic and dopaminergic interneurons in the OB [13,34,35]. While Dlx2 was widely expressed in the mantle zone of the E13.5 ventral telencephalon, only a few Dlx2^+^ cells had infiltrated the wild-type (Figure 5S) and *Neurog1*^−/−^ (Figure 5T) OBs at this stage. In contrast, Dlx2-labeled neurons were abundant in the E13.5 *Neurog1/2*^*−/−*^ OBLS (Figure 5V), lying directly adjacent to the BrdU-labeled progenitor zone in the VZ, and filling the gap between the Tbr2^+^ and BrdU^+^ zones. Some Dlx2^+^ cells were also detected in ectopic sites in the *Neurog2*^KI*/*KI^ OB (Figure 5U). Interneurons thus appeared to be generated at the expense of glutamatergic neurons in the *Neurog1/2*^*−/−*^ OBLS, and possibly also in *Neurog2*^*KI/KI*^ OBs.

**Figure 6:**
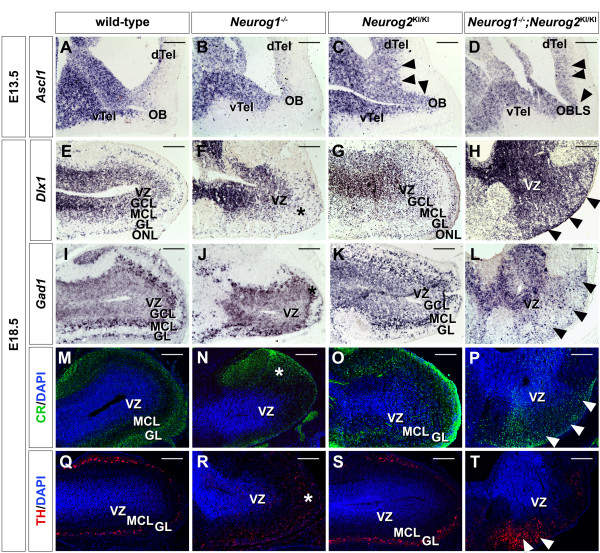
**Ectopic differentiation of olfactory bulb interneurons in *Neurog2 ***^**KI/KI **^**and *Neurog1/2 ***^**−/−**^ embryos. (**A**) to (**D**) Expression of *Ascl1* in embryonic day (**E**) 13.5 wild-type (**A**), *Neurog1*^−/−^ (**B**), *Neurog2*^KI/KI^ (**C**), and *Neurog1/2*^−/−^ (**D**) OBs. (**E**) to (**T**) Expression of *Dlx1* (**E**) to (**H**), *Gad1* (**I**) to (**L**), calretinin (CR) (**M**) to (**P**) and TH (**Q**) to (**T**) in E18.5 wild-type, (E,I,M,Q), *Neurog1*^*−/−*^ (F,J,N,R), *Neurog2*^KI/KI^ (G,K,O,S), and *Neurog1/2*^−/−^ (H,L,P,T) OBs. Blue is DAPI nuclear stain in (**M**) to (**T**). Arrowheads in (C,D) mark the upregulated expression of *Ascl1* in the OB of *Neurog2*^KI/KI^ and OBLS of *Neurog1/2*^−/−^ embryos. Arrowheads in (H,L,P,T) mark expansion of expression domain from VZ to pial surface in *Neurog1/2*^−/−^ OBs. Asterisks in (F,J,N,R) mark ectopic interneurons in *Neurog2*^KI/KI^ OBs. dTel, dorsal telencephalon; GL, glomerular layer; GCL, granule cell layer; MCL, mitral cell layer; OB, olfactory bulb; OBLS, olfactory bulb-like structure; ONL, olfactory nerve layer; vTel, ventral telencephalon; VZ, ventricular zone. Scale bars: 500 μm (**A**) to (**T**).

In the neocortex, the ventralization of *Neurog2*^*KI/KI*^ and *Neurog1/2*^*−/−*^ progenitors arises due to the increased expression of *Ascl1*[18,19], a proneural gene that is required for the generation of GABAergic neurons in the ventral telencephalon [36,37], and a subset of periglomerular cells in the embryonic OB [35] and adult OB [38]. *Ascl1* expression was also upregulated in the E13.5 OB VZ in *Neurog2*^*KI/KI*^ and *Neurog1/2*^*−/−*^ embryos (Figure 6A,B,C,D), consistent with a similar mechanism underlying the misspecification of OB neurons. To further analyze the ectopic differentiation of OB interneurons, E18.5 OBs were analyzed for the expression of *Dlx1*, which labels OB progenitors and postmitotic granule and periglomerular cells in the granule cell layer and GL, as well as glutamate decarboxylase 1 (*GAD1*), which labels all GABAergic OB interneurons in the granule cell layer and GL [39], calretinin, which labels most granule cells and a subset of periglomerular cells [6], and TH, which labels dopaminergic periglomerular cells (Figure 6 to T E) [6]. In E18.5 *Neurog1*^*−/−*^ OBs, a distinct GL was not evident, and instead, neurons labeled with *Dlx1GAD1,* and calretinin and TH were scattered throughout the mantle zone of the OB (Figure 6F,J,N,R). In E18.5 *Neurog2*^*KI/KI*^ OBs, the GL was clearly marked by *Dlx1GAD1*, and calretinin, but a scattering of ectopic interneurons labeled by these markers was also detected between the MCL and GL (Figure 6G,K,O). While TH^+^ cells were not located in ectopic sites in E18.5 *Neurog2*^*KI/KI*^ OBs, they formed a less compact layer (Figure 6S). Finally, in *Neurog1/2*^*−/−*^ OBLSs, there was a striking expansion of *Dlx1GAD1*, calretinin and TH expression domains, which spread out radially from the VZ of the OBLS to reach the pial surface of the brain (Figure 6H,L,P,T).

**Figure 7:**
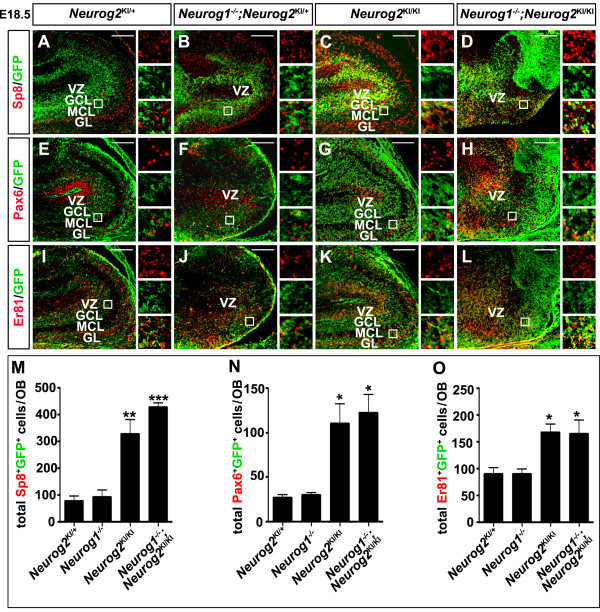
**Neuronal misspecification defects in *Neurog2***^***−/− ***^**olfactory bulbs and *Neurog1/2 ***^***−/− ***^**olfactory bulb-like structures.** (**A**) to (**L**) Co-immunostaining of GFP (green, **A** to **L**) with Sp8 (red, **A** to **D**), Pax6 (red, **E** to **H**) or Er81 (red, **I** to **L**) in E18.5 wild-type, (**A**,**E**,**I**), *Neurog1*^*−/−*^ (**B**,**F**,**J**), *Neurog2*^KI/KI^ (C,G,K), and *Neurog1/2*^−/−^ (**D**,**H**,**L**) OBs. Insets to the right of each panel are fourfold magnifications of boxed areas. (**M**) to (**O**) Quantitation of total Sp8^+^GFP^+^ cells (**M**), Pax6^+^GFP^+^ cells (**N**), and Er81^+^GFP^+^ cells (**O**) in embryonic day 18.5 wild-type, *Neurog1*^*−/−*^, *Neurog2*^KI/KI^ and *Neurog1/2*^−/−^ OBs. *P <0.05, **P <0.01, ***P <0.005. GCL, granule cell layer; GL, glomerular layer; MCL, mitral cell layer; VZ, ventricular zone. Scale bars: 500 μm (**A**) to (**L**).

Aberrantly positioned OB interneurons could signify an increase in migration from ventral domains or a respecification of dorsal progenitors to acquire an aberrant ventral identity. While our previous results in the neocortex favor a respecification model, to formally test this, we performed *Neurog2* short-term lineage tracing with the *Neurog2*^*GFP*KI^ allele. Note that we have extensively compared molecular marker expression in wild-type and *Neurog2*^KI/+^ brains (including the OB) and have no evidence for a heterozygous phenotype. We therefore analyzed GFP co-expression with three transcription factors expressed in OB interneuron populations [40]; namely Sp8, which is expressed in calretinin^+^, parvalbumin^+^ and GABAergic periglomerular and granule cells [41,42]; Pax6, which is required to generate the majority of granule cells along with dopaminergic (TH^+^) periglomerular cell progenitor subtypes [43,44]; and Er81, which labels VZ progenitors, granule cells and dopaminergic periglomerular cells [45]. As expected, minimal GFP/Sp8 co-labeling was detected in E18.5 *Neurog2*^KI/+^ (that is, wild-type control; Figure 7A,M) and *Neurog2*^*KI/*+^;*Neurog1*^−/−^ OBs (Figure 7B,M). In contrast, in E18.5 *Neurog2*^KI/KI^ OBs (4.2-fold increase; *P* <0.001; Figure 7C,M) and *Neurog1/2*^*−/−*^ OBLSs (5.5-fold increase; *P* <0.0001; Figure 7D,M) there was a significant increase in number of GFP/Sp8 co-labeled cells throughout the OB. Similarly, the numbers of Pax6^+^GFP^+^ (Figure 7E,F,G,H,N) and Er81^+^GFP^+^ (Figure 7I,J,K,L,O) double-positive interneurons were also significantly higher in *Neurog2*^*KI/KI*^ OBs (Pax6^+^GFP^+^: 4.07-fold increase, *P* <0.05; Er81^+^GFP^+^: 1.87-fold increase, *P* <0.05) and *Neurog1/2*^*−/−*^ OBLSs (Pax6^+^GFP^+^: 4.52-fold increase, *P* <0.05; Er81^+^GFP^+^: 1.83-fold increase, *P* <0.05).

In *Neurog2*^*KI/KI*^ OB and *Neurog1/2*^*−/−*^ OBLSs, therefore, a subset of pallial progenitors that should give rise to glutamatergic OB projection neurons are misspecified, instead differentiating into GABAergic interneurons. In contrast, neuronal misspecification defects are not observed in *Neurog1*^*−/−*^ OBs, although the migration of GABAergic OB neurons is strikingly perturbed.

### Olfactory sensory neurons fail to innervate the olfactory bulb in *Neurog1*^*−/−*^ and *Neurog1/2*^*−/−*^ embryos

At first glance, the defective migration of OB interneurons in *Neurog1*^*−/−*^ and *Neurog1/2*^*−/−*^ embryos was unexpected, given that these proneural genes are not expressed in OB interneuron lineages [20]. However, several studies have indicated that OSN innervation is required for OB interneuron migration [13-16], in addition to controlling the proliferation of OB progenitors [11]. Defects in OB interneuron migration could thus be non-cell autonomous in *Neurog1*^*−/−*^ and *Neurog1/2*^*−/−*^ double mutants. Consistent with this model, *Neurog1* is expressed in OE progenitors, where it is required for the differentiation of a subset of OSNs at early stages of development [22], although innervation patterns were not examined.

**Figure 8:**
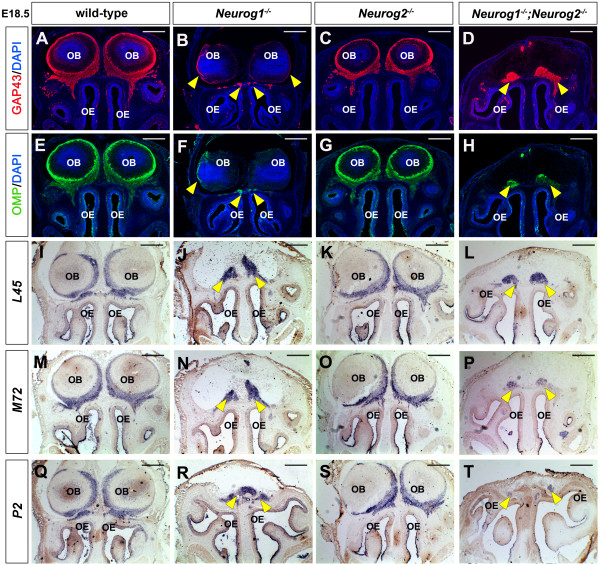
***Neurog1 ***^***−/− ***^**and *Neurog1/2 ***^***−/− ***^**olfactory sensory neurons express mature markers but fail to innervate olfactory bulb.** (**A**) to (**H**) Coronal sections of the embryonic day (E) 18.5 OB showing expression of GAP43 (red, **A** to **D**) and OMP (green, **E** to **H**) with DAPI (blue, **A** to **H**) in wild-type (**A**,**E**), *Neurog1*^*−/−*^ (**B**,**F**), *Neurog2*^KI/KI^ (**C**,**G**) and *Neurog1/2*^−/−^ (**D**,**H**) embryos. Yellow arrows denote loss of OSN innervation of the OB in *Neurog1*^*−/−*^ and *Neurog1/2*^−/−^ (**B**,**D**,**F**,**H**) embryos. **(I) to (T)** Expression of odorant receptors *L45* (**I**) to (**L**), *M72* (**M**) to (**P**) and *P2* (**Q**) to (**T**) in E18.5 wild-type (**I**,**M**,**Q**), *Neurog1*^*−/−*^ (**J**,**N**,**R**), *Neurog2*^KI/KI^ (**K**,**O**,**S**) and *Neurog1/2*^−/−^ (**L**,**P**,**T**) embryos. Note that the lack of an apparent OB in *Neurog1*^−/−^ sections is because the OB is shortened along the proximodistal axis, and hence does not fill the rostral-most part of the cavity in the skull, where OR expression levels are the highest (**J**,**N**,**R**). GAP43, growth-associated protein 43; OB, olfactory bulb; OE, olfactory epithelium; OMP, olfactory marker protein; OSN, olfactory sensory neuron. Scale bars: 500 μm (**A**) to (**T**).

**Figure 9:**
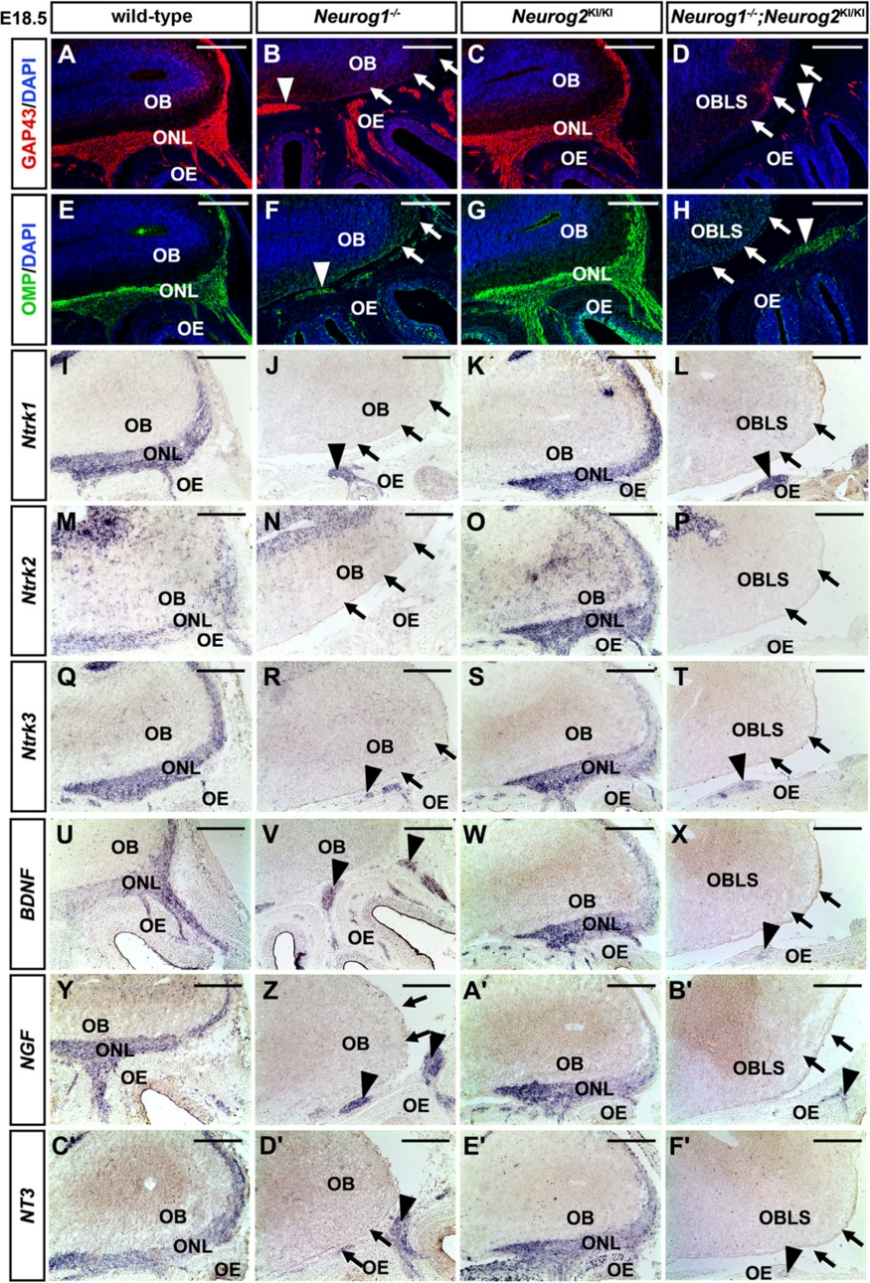
***Neurog1 ***^***−/− ***^**and *Neurog1/2 ***^***−/− ***^**olfactory sensory neurons express neurotrophic receptors and ligands but fail to innervate olfactory bulb.** (**A**) to (**H**) Expression of GAP43 (red, **A** to **D**) and OMP (green, **E** to **H**) with DAPI counterstain (blue, **A** to **H**) in embryonic day (E) 18.5 wild-type (**A**,**E**), *Neurog1*^*−/−*^ (**B**,**F**), *Neurog2*^KI/KI^ (**C**,**G**) and *Neurog1/2*^−/−^ (**D**,**H**) OBs/OEs. White arrows denote loss of synaptogenesis between the OE and the OB in *Neurog1*^*−/−*^ and *Neurog1/2*^−/−^ (**B**,**D**,**F**,**H**) olfactory systems, and arrowheads point to the accumulation of OSN axons in a FCM. (**I**) to (**T**) Expression of *Ntrk1* (**I**) to (**L**), *Ntrk2* (**M**) to (**P**) and *Ntrk3* (**Q**) to (**T**) in E18.5 wild-type (**I**,**M**,**Q**), *Neurog1*^*−/−*^ (**J**,**N**,**R**), *Neurog2*^KI/KI^ (**K**,**O**,**S**) and *Neurog1/2*^−/−^ (**L**,**P**,**T**) embryos. (**U**) to (**F'**) Expression of *BDNF* (**U**) to (**X**), *NGF* (**Y**) to (**B'**), *NT3* (**C'**) to (**F'**), in E18.5 wild-type (**U**,**Y**,**C'**), *Neurog1*^*−/−*^ (**V**,**Z**,**D'**), *Neurog2*^KI/KI^ (**W**,**A'**,**E'**) and *Neurog1/2*^−/−^ (**X**,**B'**,**F'**) embryos. Black arrows (**J**,**L**,**N**,**P**,**R**,**T**,**V**,**X**,**Z**,**B'**,**D'**,**F'**) indicate loss of marker expression in outer layers of *Neurog1*^*−/−*^ and *Neurog1/2*^−/−^ OBs. Arrowheads (**J**,**L**,**R**,**T**,**V**,**X**,**Z**,**B'**,**D'**,**F'**) point to FCM formation in *Neurog1*^*−/−*^ and *Neurog1/2*^−/−^ embryos. FCM, fibrocellular mass; OB, olfactory bulb; OBLS, olfactory bulb-like structure; OE, olfactory epithelium; ONL, olfactory nerve layer. Scale bars: 500 μm (**A**) to (**Z**), (**A'**) to (**F'**).

To determine whether OSN innervation was indeed perturbed in the absence of *Neurog1* function, we monitored the expression of growth-associated protein 43 (GAP43) and olfactory marker protein (OMP), which mark both the cell bodies and axonal projections of immature (GAP43) and mature (OMP) OSNs [11,46]. In coronal sections through E18.5 wild-type (Figure 8A,E) and *Neurog2*^*KI/KI*^ (Figure 8C,G) OBs, GAP43-labeled and OMP-labeled OSN axons emanated from the OE, traversing the cribriform plate to penetrate the ONL, where they wrapped the entire periphery of the OB. In contrast, in E18.5 *Neurog1*^*−/−*^ (Figure 8B,F) and *Neurog1/2*^*−/−*^ (Figure 8D,H) embryos, GAP43 and OMP labeled a fibrocellular mass (FCM) that did not penetrate the OB. Only a small amount of GAP43 and OMP expression was observed surrounding caudal regions of the *Neurog1*^*−/−*^ OB, suggesting that very few OSN axons innervated the mutant OB (Figure 8B,F). As a side note, the term FCM was first coined to describe the *extratoes* (that is, *Gli3*^−/−^) olfactory phenotype, and refers to an amorphous bundle of OSN axons that fail to extend and penetrate the OB [47]. To assess OSN innervation along the entire rostrocaudal axis, we also examined sagittal sections of E18.5 *Neurog1*^*−/−*^ (Figure 9B,F) and *Neurog1/2*^*−/−*^ (Figure 9D,H) embryos with calretinin (data not shown), GAP43 (Figure 9A,B,C,D) and OMP (Figure 9E,F,G,H), revealing that defects in OSN axon innervations of the OB were observed at all levels.

OSNs express one of ~1,200 odorant receptors (OR) in mice, dictating the type of odor they will respond to, with OSNs that express the same OR targeting the identical glomerulus in the OB [48-50]. Notably, the specificity of OSN targeting depends on ORs, which are functionally required to establish a glomerular topographic map in the OB [51-53]. To determine whether OR expression was maintained in *Neurog1/2*^*−/−*^ OSNs, we examined the expression of three different ORs (*L45M72P2*) that direct the innervation of distinct glomeruli [54,55]. In coronal sections through E18.5 wild-type OBs (Figure 8I,M,Q) and *Neurog2*^*KI/KI*^ OBs (Figure 8K,O,S), *L45M72* and *P2* transcripts were detected in OSN axon bundles that had innervated the OB, concentrating in the ventromedial ONL. In contrast, in E18.5 *Neurog1*^*−/−*^ embryos (Figure 8J,N,R) and *Neurog1/2*^*−/−*^ embryos (Figure 8L,P,T), *L45M72* and *P2* were expressed in OSN axons that accumulated in a FCM outside the OB. *Neurog1*^*−/−*^ and *Neurog1/2*^*−/−*^ OSN axons therefore failed to penetrate the OB, even though they continued to express ORs.

We next searched for molecular signals that may account for the lack of OSN innervation in *Neurog1*^*−/−*^ and *Neurog1/2*^*−/−*^ OBs. Neurotrophins (*NGFBDNFNT3*) and their cognate receptors (*Ntrk1Ntrk2Ntrk3*) regulate several cellular processes, including neuronal survival, differentiation and axonal and dendritic growth (reviewed in [56]). The ligands *BDNFNGF* and *NT3* and the receptors *Ntrk1Ntrk2* and *Ntrk3* are all expressed in the olfactory system reviewed in [56]. In sagittal sections through E18.5 wild-type OBs/OEs (Figure 9I,M,Q,U,Y,C') and *Neurog2*^*KI/KI*^ OBs/OEs (Figure 9K,O,S,W,A',E'), *BDNFNGFNT3Ntrk1Ntrk2* and *Ntrk3* were all expressed in a similar fashion, marking OSN axons exiting the OE and innervating the ONL of the OB. In contrast, in E18.5 *Neurog1*^*−/−*^ embryos (Figure 9J,N,R,V,Z,D') and *Neurog1/2*^*−/−*^ embryos (Figure 9L,P,T,X,B',F'), *BDNFNGFNT3Ntrk1Ntrk2* and *Ntrk3* transcripts accumulated in FCMs between the OB and OE, consistent with the inability of OSNs to innervate the OB in these two mutant backgrounds.

Taken together, these data show that *Neurog1*^*−/−*^ and *Neurog1/2*^*−/−*^ mutant OSNs fail to innervate the OB, despite their expression of several markers of differentiated OSNs. *Neurog1* is thus required to promote OSN axonal extension into the ONL of the OB.

### Olfactory sensory neurons and olfactory ensheathing cells express appropriate differentiation markers in *Neurog1*^*−/−*^ and *Neurog1/2*^*−/−*^ embryos

**Figure 10:**
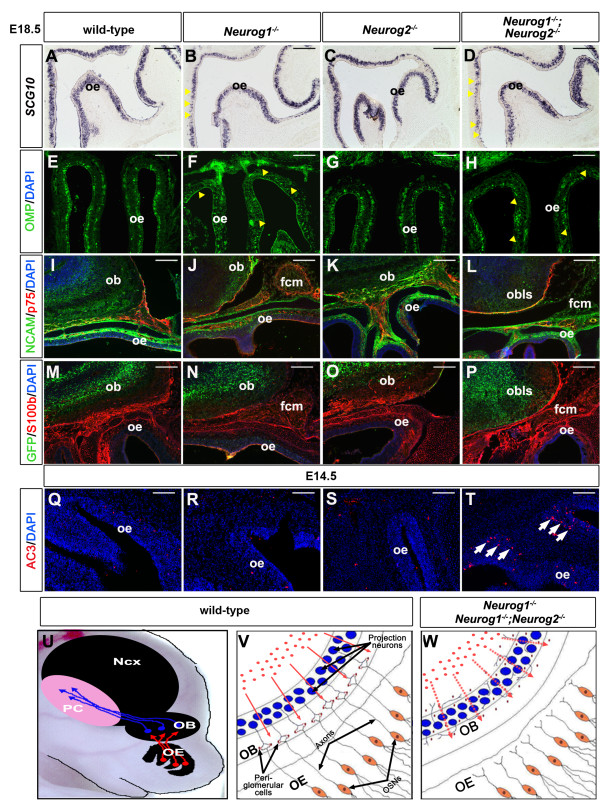
**Normal OSN and OEC differentiation in *Neurog1 ***^***−/− ***^**and *Neurog2 ***^***−/− ***^**mutants, but apoptosis elevated in double mutants.** (**A**) to (**D**) Expression of *SCG10* in wild-type (**A**), *Neurog1*^*−/−*^ (**B**), *Neurog2*^−/−^ (**C**), and *Neurog1/2*^−/−^ (**D**) OBs at embryonic day (**E**) 18.5. (**E**) to (**P**) Co-labeling with DAPI (blue, **E** to **P**) and OMP (green, **E** to **H**), NCAM (green, **I** to **L**), GFP (green, **M** to **P**), p75 (red, **I** to **L**), or S100b (red, **M** to **P**) in wild-type (**E**,**I**,**M**), *Neurog1*^*−/−*^ (**F**,**J**,**N**), *Neurog2*^−/−^ (**G**,**K**,**O**), and *Neurog1/2*^−/−^ (**H**,**L**,**P**) OBs at E18.5. (**Q**) to (**T**) Co-labeling with DAPI (blue) and activated caspase 3 (AC3; red) in wild-type (**Q**), *Neurog1*^*−/−*^ (**R**), *Neurog2*^−/−^ (**S**), and *Neurog1/2*^−/−^ (**T**) OBs at E14.5. (**U**) to (**W**) Schematic representation of the three components of the olfactory system (**U**). The normal process of OSN innervation of the OB, and the subsequent formation of the glomerular layer by migrating OB interneurons (**V**) is perturbed in *Neurog1/2*^*−/−*^ embryos (**W**). OB, olfactory bulb; OE, olfactory epithelium; OEC, olfactory ensheathing cell; OSN, olfactory sensory neuron; PC, piriform cortex. Scale bars: 500 μm (**A**) to (**P**), 250 μm (**Q**) to (**T**).

To better understand why *Neurog1*^*−/−*^ and *Neurog1/2*^*−/−*^ OSN axons did not penetrate the OB/OBLS, we examined the OE in more detail. In a previous report, it was shown that fewer OSNs express a subset of differentiation markers in the E12.5 *Neurog1*^*−/−*^ OEs [22]. Here we examined OSN differentiation at E18.5, using the pan-neuronal marker *SCG10* (Figure 10A,B,C,D) and the mature OSN marker OMP (Figure 10E,F,G,H). Strikingly, there was only a slight reduction in the number of *SCG10*-labeled and OMP-labeled OSNs in medial domains of the E18.5 *Neurog1*^*−/−*^ OEs (Figure 10B,F) and *Neurog1/2*^*−/−*^ OEs (Figure 10D,H) compared with E18.5 wild-type embryos (Figure 10A,E) and *Neurog2*^*KI/KI*^ embryos (Figure 10C,G). A significant number of OSNs thus differentiate by E18.5 in *Neurog1*^*−/−*^ and *Neurog1/2*^*−/−*^ OEs, despite the earlier block in differentiation [22].

We next examined the differentiation of olfactory ensheathing cells (OECs), which arise in the olfactory placode and wrap around OSN axonal tracts to provide trophic support and promote OSN axonal growth [57,58]. NCAM, which is expressed in OSNs and OECs, and p75, which specifically labels OECs, co-labeled the olfactory nerve, which infiltrated the ventral surface of the OB in E18.5 wild-type (Figure 10I) and *Neurog2*^*KI/KI*^ (Figure 10K) OEs. In contrast, while NCAM and p75 were co-expressed in the olfactory nerve in E18.5 *Neurog1*^−/−^ embryos (Figure 10J) and *Neurog1/2*^−/−^ embryos (Figure 10L), the OEC-wrapped OSNs terminated in a FCM between the OB and OE. Labeling of OECs with S100b similarly revealed that OECs infiltrate the ventral OB in wild-type embryos (Figure 10M) and *Neurog2*^*KI/KI*^ embryos (Figure 10O), whereas OECs accumulate in a FCM in *Neurog1*^*−/−*^ embryos (Figure 10N) and *Neurog1/2*^*−/−*^ embryos (Figure 10P). OSNs and OECs thus differentiate in all *Neurog1/2* genotypes, but they fail to innervate the OB in *Neurog1*^*−/−*^ and *Neurog1/2*^*−/−*^ embryos.

Finally, we investigated whether apoptosis may contribute to the small decline in OSN numbers in *Neurog1*^*−/−*^ and *Neurog1/2*^*−/−*^ mutants by analyzing the expression of activated caspase 3, a marker of apoptosis. In E14.5 wild-type OEs (Figure 10Q), *Neurog1*^*−/−*^ OEs (Figure 10R) and *Neurog2*^*KI/KI*^ OEs (Figure 10S), only a few scattered activated caspase 3-positive cells were detected, whereas in *Neurog1/2*^*−/−*^ embryos (Figure 10T) there was a notable increase in activated caspase 3 immunolabeling in the OE. Apoptosis thus occurs at elevated levels in the *Neurog1/2*^*−/−*^ OE only, despite *Neurog2* not being expressed in the vast majority of OE progenitors. Strikingly, the increase in OE apoptosis in double mutants phenocopies the OE defects observed upon bulbectomy [59], suggesting that the *Neurog1/2*^*−/−*^ OBLS may fail to provide trophic signals to the OE, as discussed further below.

## Discussion

The olfactory system consists of the OB, OE and olfactory cortex, which together are responsible for detecting and processing odors (Figure 10U). Here we provide mechanistic insights into how the development of these olfactory structures is coordinated. We first demonstrate that *Neurog1* and *Neurog2* function redundantly and in a cell autonomous fashion to specify the glutamatergic neuronal identity of OB projection neurons and juxtaglomerular cells, while suppressing an alternative interneuron fate. In contrast, only *Neurog1* is required to regulate OSN innervation of the OB, defects in which can perturb the proliferation rate of OB progenitors, and the migratory routes of OB neurons (Figure 10V,W). In summary, *Neurog1* and *Neurog2* play an integral role in coordinately regulating development of the olfactory system, regulating cell fate specification in the OB and OSN differentiation and axonal targeting in the OE.

### *Neurog1/2* promote a glutamatergic neuronal identity in the olfactory bulb

Glutamatergic mitral, tufted and juxtaglomerular cells are derived from dorsal telencephalic progenitors, as revealed by *Neurog1*[20] and *Neurog2* (present study and [1]) lineage tracing. Accordingly, we found that fewer glutamatergic OB neurons are generated in the absence of *Neurog1/2* function. Nevertheless, a subset of mitral and juxtaglomerular cells differentiate in the *Neurog1/2*^*−/−*^ OBLS, suggesting that other genes compensate for the loss of proneural function. Candidate transcriptional regulators that may promote the differentiation of glutamatergic OB neurons in the absence of *Neurog1/2* include the cortical selector genes *Pax6*[3,60] and *Lhx2*[61], both of which are also required for the differentiation of subsets of glutamatergic neuronal lineages in the OB. Consistent with a potential compensatory role for *Pax6* in the OB, in the embryonic neocortex, we previously demonstrated that *Neurog1/2* are required for the first wave of neurogenesis (<E14.5), whereas *Pax6* drives the second wave (>E14.5) [19].

At first glance, the presence of OB defects in *Neurog1*^*−/−*^ and not *Neurog2*^*−/−*^ single mutants might suggest that these two transcription factors have distinct functions. However, we show here that *Neurog1* is upregulated in the presumptive OB of *Neurog2*^*−/−*^ single mutants, probably compensating for the loss of *Neurog2.* We thus suggest that *Neurog1* and *Neurog2* are for the most part functionally redundant in the developing OB. Consistent with this idea, only in *Neurog1/2*^*−/−*^ double mutants are severe defects in OB development observed.

OB and neocortical projection neurons differ, yet both arise from adjacent pools of dorsal telencephalic progenitors. How does neuronal diversification occur? One possibility is that OSN-derived or OEC-derived signals alter the cell-fate specification functions of *Neurog1/2*. Consistent with this idea, at ~ E11 when mitral cells begin to differentiate, OSN pioneer axons infiltrate the primordial OB [10,11], as do OECs, which wrap OSN axons [57,62-65]. How might OSNs/OECs influence the cell-fate specification properties of *Neurog1/2* in the OB? OSNs secrete Fgf8 to noncell-autonomously reduce OB progenitor cell proliferation [11-16], while OECs produce an unknown chemoattractant that guides OB neuronal migration [66]. One possibility is that the activation of downstream signaling pathways in the OB triggers a change in the cell-fate specification properties of *Neurog1* and *Neurog2.* For instance, modification by Neurog1/2 by phosphorylation might result in the capacity of these proneural genes to turn on the expression of genes such as *Tcfap2e*, which is specifically expressed in OB lineages, a possibility that will be investigated in the future.

### *Neurog1/2* control a binary choice between excitatory and inhibitory lineages in the olfactory bulb

In the region of the dorsal telencephalon that will become the neocortex, *Neurog1/2* regulate a binary fate choice between dorsal, glutamatergic versus ventral, GABAergic neuronal fates [18,19]. Consequently, in *Neurog2*^*KI/KI*^ and *Neurog1/2*^*−/−*^ embryos, neocortical progenitors and their neuronal derivatives are misspecified, acquiring a dorsal LGE-like identity [19]. Notably, the dorsal LGE is the ventral telencephalic progenitor zone from which most OB interneurons arise during embryogenesis, including granule cells and periglomerular cells [13,35,43,44,67]. Consistent with the expansion of a dorsal LGE-like progenitor pool in *Neurog2*^*KI/KI*^ and *Neurog1/2*^*−/−*^ embryos, several interneuron markers were ectopically expressed in the mutant OBs/OBLSs.

OB interneuron differentiation is regulated by multiple transcription factors, including *Ascl1* and *Dlx1/2*, which control distinct differentiation pathways [6,35,68]. Here we found that *Ascl1* and *Dlx1/2* are both upregulated in the *Neurog2*^*KI/KI*^ OB and *Neurog1/2*^*−/−*^ OBLS from E13.5 of development, as previously reported in the neocortex [18]. Additional transcription factors required for the differentiation of subsets of OB interneurons were also ectopically expressed in the *Neurog2*^*KI/KI*^ OB and *Neurog1/2*^*−/−*^ OBLS, including *Sp8*[41], *Pax6*[44,69] and *Er81*[70]. By monitoring interneuron marker expression in GFP-labeled OB cells derived from the *Neurog2* lineage, we were able to show that the ectopic Sp8-expressing, Pax6-expressing and Er81-expressing interneurons in *Neurog2*^*KI/KI*^ and *Neurog1/2*^*−/−*^ OBs were derived from pallial progenitors that had undergone a fate switch, as opposed to an increase in the migration of OB interneurons. *Neurog1/2* thus play a similar role in regulating a binary fate switch between an excitatory glutamatergic neuronal phenotype versus inhibitory interneuron phenotype in both the OB (present study) and neocortex [18,19].

### *Neurog1* regulates OB tissue morphogenesis, proliferation and lamination by controlling OSN innervation of the OB

We show here that *Neurog1/2*^*−/−*^ embryos have severe defects in OB morphogenesis, forming an aberrantly localized OBLS in the ventrolateral brain. Interestingly, similar OB morphological defects are also observed in *Pax6* mutants [3,60] and *Lhx2* mutants [71], cortical selector genes that are required to specify dorsal telencephalic regional identities [72,73]. In contrast, the morphogenetic defects observed in the *Neurog1*^*−/−*^ OB are more modest, with a reduction in OB size and aberrant lamination of the GL and MCL. Given that the driving force for OB morphogenesis is thought to be a reduction in proliferation in the presumptive OB at the rostral tip of the telencephalon, which is left behind as surrounding neocortical territories expand [11,12], we examined proliferation in *Neurog1/2* mutant embryos. We found that proliferation rates do not decline in the presumptive OB versus neocortex in either *Neurog1*^*−/−*^ or *Neurog1/2*^*−/−*^ embryos, probably accounting at least in part for the inability of the OB to protrude outwards. Nevertheless, differences in proliferation alone cannot explain why the OBLS morphogenesis defects in *Neurog1/2*^*−/−*^ embryos are so much more striking than those observed in *Neurog1*^*−/−*^ OBs. We speculate that the added OB neuronal specification defects observed in *Neurog1/2*^*−/−*^ embryos (present study), *Pax6*^*−/−*^ embryos [3,60] and *Lhx2*^*−/−*^ embryos [71], which are not observed in the *Neurog1*^*−/−*^ OB, may alter neuronal migratory routes, hence influencing the aberrant positioning of the OBLS.

Several studies have suggested that the normal reduction in proliferation of presumptive OB versus neocortical progenitors is induced by the innervation of the OB by OSN axons [11]. The first pioneer OSN axons innervate the OB at E11, when OB morphogenesis first begins, but it is not until E13 to E15 that a sizeable number of OSN axons enter the OB, first innervating the ONL and later infiltrating the GL, where they make synaptic contacts with mitral cell dendrites [11,64,74-76]. Consistent with these studies, we found that *Neurog1*^*−/−*^ and *Neurog1/2*^*−/−*^ OSN axons do not innervate the OB, instead terminating prematurely in a FCM. The FCM formation and lack of OB innervation in *Neurog1*^−/−^ embryos is also strikingly similar to the phenotypes observed in *ArxFezf1* and *Dlx5* mutants, which also develop a smaller OB with aberrant MCL and GL lamination [13-16]. However, *Dlx5Arx* and *Fezf1* are not expressed in pallial lineages – but rather in subpallial and/or OSN lineages, where they control the differentiation and/or migration of OB interneurons through cell autonomous and nonautonomous mechanisms [13-16]. Strikingly, the abnormal formation of the MCL and GL in *Neurog1*^−/−^ OBs more closely resembles phenotypes observed following the mutation of genes that are expressed in OSN lineages and prevent innervations of the OB, including *Fezf1Dlx5* and *Klf7*[16,77,78]*.*

Why do *Neurog1*^*−/−*^ OSNs fail to innervate the OB? The basal lamina surrounding the brain is remodeled at E14.5 to allow OSN axon penetration, an event that depends on canonical Wnt signaling [79] and matrix metalloproteinases. In *Dlx5* mutants, the defects in OSN penetration of the OB may be related either to defective differentiation of OSNs, which similar to *Neurog1*^*−/−*^ OSNs also express markers of differentiated neurons, or in the frontronasal mesenchyme, which also expresses *Dlx5*[13]. In *Fezf1* mutants, the removal of this basal lamina has been shown to rescue the OSN phenotype, resulting in OSN penetration of the OB. Other possibilities include the loss of a chemoattractant activity in the *Neurog1*^*−/−*^ OB itself. While we did not identify any defects in the expression of the neurotrophin receptors or ligands in the OB or OE of *Neurog1/2*^*−/−*^*Neurog1* has been shown to regulate the OB expression of prokineticin 2 (PK2) [21], a secreted proteins that binds G-protein coupled receptors. Notably, *PK2*-deficient mice phenocopy the *Neurog1*^*−/−*^ OB defects, at least in part because PK2 functions as a chemoattractant for OB interneurons born in the ventral telencephalon [80]. Future work will be required to determine whether PK2 also functions as a chemoattractant for OSN axons, and to determine whether *Neurog1* function is required in the OB and/or OE for OSN innervation of the OB.

### *Neurog1* is required for olfactory sensory neuron innervation of the olfactory bulb

Previous analyses of the *Neurog1*^*−/−*^ OE revealed that fewer OSNs express a subset of mature neuronal markers at early developmental stages (E12.5), including the pan-neuronal marker *SCG10*, suggestive of a block in differentiation [22]. However, these defects are only partial, as other OSN markers, such as *Ebf1* and *Lhx2*, are expressed at normal levels in E12.5 *Neurog1*^*−/−*^ OSNs. Here we examined the differentiation of *Neurog1*^*−/−*^ and *Neurog1/2*^−/−^ OSNs at a later developmental stage (E18.5), revealing only a minor reduction in the expression of mature OSN markers, including *SCG10*, GAP43, OMP and the OR genes *L45M72* and *P2*. The expression of mature OSN markers in the *Neurog1*^*−/−*^ OE may be due in part to the maintained expression of *Lhx2*, which is required to initiate OE differentiation [61], or *Six1*, which functions upstream of *Neurog1* to regulate OSN differentiation [81].

In addition to the ability of the OE to influence OB development, it has conversely been suggested that the OB can influence the OE. Indeed, bulbectomy results in a loss of OSN marker expression and increased apoptosis in the OE [59]. In this regard it is interesting that in *Neurog1/2*^*−/−*^ OE there is an increase in apoptosis that is not observed in the *Neurog1*^*−/−*^ OE. At first glance, this is surprising, as *Neurog2* is only expressed in a small dorsomedial domain of the OE (present study), whereas *Neurog1* expression is widespread [22]. While *Neurog2* cannot rescue the OSN innervation defects observed in *Neurog1*^*−/−*^ embryos, we cannot rule out the possibility that *Neurog2* initiates the expression of survival signals in the OSN, thereby compensating for the loss of *Neurog1* in the OE. However, given the limited expression domain of *Neurog2,* we do not believe that this is the case. Instead we suggest that the *Neurog1/2*^*−/−*^ OBLS is deficient in a trophic signal that is an essential survival signal for OSNs in the OE. While we investigated whether the neurotrophins might be contributing to the death of the OSNs, no defects in Ntrk receptor or ligand expression in the OE was observed in the *Neurog1/2* mutants, suggesting that other factors must be involved.

## Conclusions

In this article we find that both *Neurog1* and *Neurog2* are expressed in OB progenitors, where they function redundantly to specify the identities of glutamatergic OB neurons, including mitral and juxtaglomerular cells. Conversely we show that *Neurog1* is required to promote OSN innervation of the OB, and consequently influences OB proliferation and morphogenesis. We thus conclude that the proneural genes *Neurog1* and *Neurog2* coordinately regulate development of the olfactory system by regulating proliferation, cell fate specification, neuronal migration and axonal innervation.

## Methods

### Animals and genotyping

The generation of *Neurog1* and a *Neurog2*^*GFP*^ KI null allele was previously described [24,29]. Double heterozygous mice carrying null alleles of *Neurog1* and *Neurog2*^*KI*^ were maintained on a CD1 background and males and females were crossed to generate embryos. Mating was confirmed via vaginal plugs, with mouse embryos being staged by considering the plug date as E0.5. Embryos were genotyped as previously described [19,24]. All animal procedures were approved by the University of Calgary Animal Care Committee (Protocol # AC11-0053) in agreement with the Guidelines of the Canadian Council of Animal Care (CCAC).

### RNA *in situ* hybridization

Embryonic dissections were performed in PBS. Tissue was fixed at 4°C in 4% paraformaldehyde/1× PBS overnight, rinsed in 1× PBS and then cryoprotected in 20% sucrose/1× PBS at 4°C before embedding in Tissue-Tek optimum cutting temperature (O.C.T.) compound (VWR Canada, Mississauga, ON, Canada). Tissue sections (10 μm) were collected on Superfrost Plus (Fisher Scientific, Ottawa, ON, Canada) slides using a CM3050-S cryostat (Leica Microsystems, Richmond Hill, ON, Canada). RNA *in situ* hybridization was performed as previously described [82]. The following riboprobes were used: *Ascl1*[83], *BDNF* (IMAGE:1397218), *Dlx1*[84], *Emx1*[85], *Fgfr1*[86], *Gad1*[87], *Lhx2*[72], *NeuroD1*[88], *NeuroD6*[89], *Neurog1* (IMAGE:30146192), *Neurog2*[17], *NGF* (IMAGE: 4190781), *NT3* (IMAGE: 1177923), *Ntrk1* (IMAGE: 421391), *Ntrk2* (IMAGE:5707891), *Ntrk3* (IMAGE: 40110345), *SCG10*[90], *Tcfap2e* (IMAGE:778986) and *L45M72* and *P2* odorant receptors (gifts from J-F Cloutier).

### Immunohistochemistry

Tissue sections were blocked in 10% horse serum/1× TBST (50 mM Tris–HCl, 150 mM NaCl, pH 7.4, 0.05% Triton X-100) for 1 hour at 37°C. For BrdU immunolabeling, the sections were first denatured in 2 N HCl for 30 minutes at 37°C prior to applying the blocking solution. Sections were incubated with primary antibodies diluted in 1× TBST overnight at 4°C. Sections were washed three times in 1× TBST for 10 minutes, then incubated with secondary antibodies diluted in 1× TBST for 1 hour at room temperature, before washing the sections again three times with 1× TBST for 10 minutes. Next, DAPI (Polysciences Inc., Warrington PA, USA) diluted in 1× TBST (1/10,000) was applied for 5 minutes. Finally, the sections were washed three times in 1× TBST, mounted with Aqua Polymount (Polysciences). The following primary antibodies were used: rabbit anti-activated caspase 3 (1/500; Promega, Madison, WI, USA), rat anti-Brdu (1/500; Roche, Mississauga, ON, Canada), rabbit anti-calretinin (1/2,000; Swant, Bellinzona, Switzerland), rabbit anti-Er81 (1/300; Developmental Studies Hybridoma Bank, Iowa City, IA, USA), rabbit anti-GAP43 (1/750; Millipore Canada, Etobicoke, ON, Canada), rabbit anti-GFP (1/500; Invitrogen (Burlington, ON, Canada), mouse anti-GFP (1/500; Millipore), rat anti-L1 (1/500; Millipore), goat anti-Neurog1 (1/100; Santa Cruz Biotechnology Inc., Santa Cruz, CA, USA), mouse anti-Neurog2 (1/3; Dr David Anderson [91]), goat anti-OMP (1/5,000; Wako Chemicals USA, Richmond, VA, USA), rabbit anti-Pax6 (1/500; Berkeley Antibody Company (BAbCO, Richmond, CA, USA), rat anti-PSA-NCAM (1/500; Millipore,), rabbit anti-S100b (1/500; Dako Canada Inc., Burlington, ON, Canada), rabbit anti-Slc7a6 (1/600; Synaptic Systems GmbH, Goettingen, Germany), rabbit anti-Slc17a7 (1/750; Synaptic Systems), goat anti-Sp8 (1/1,000; Santa Cruz), rabbit anti-TH (1/500; Santa Cruz), rabbit anti-Tbr1 (1/600; AbCam (Cambridge, MA, USA), rabbit anti-Tbr2 (1/800; AbCam), rabbit anti-vGlut1 (1/500; Synaptic Systems) and rabbit anti-vGlut2 (1/500; Synaptic Systems).

### Histological staining

Whole E18.5 heads were placed in Bouin’s fixative and processed for paraffin sectioning as previously described [92]. Sections were deparaffinized in three xylene washes for 3 minutes each, followed by rehydration in a decreasing ethanol series (2× 100%, 2× 95% and 2× 80%) for 3 minutes each. Slides were then immersed in water for 5 minutes, before staining in hematoxylin for 3 minutes. The slides were then rinsed in water for 2 minutes, and stained in eosin for 30 seconds. Slides were then dehydrated in 3-minute ethanol washes in an ascending series (2× 80%, 2× 95% and 2× 100%). Finally, the tissues were incubated in xylene overnight, and mounted in Permount SP15-100 Toluene Solution (Fisher Scientific).

### Statistical analysis

Composite photomicrographs of the entire OB were used to count immunoreactive cells from a minimum of three embryos and three sections per embryo. Graphs and statistical tests were generated with GraphPad Prism Software version 5.0 (GraphPad Software Inc., La Jolla, CA, USA). Error bars represent the standard error of the mean. Statistical significance was determined using one-way analysis of variance and a *post-hoc* Tukey’s test.

## Abbreviations

AON: accessory olfactory nucleus; DAPI: 4′,6-diamidino-2-phenylindole; E: embryonic day; FCM: fibrocellular mass; GAD1: glutamate decarboxylase 1; GAP43: growth-associated protein 43; GFP: green fluorescent protein; GL: glomerular layer; H & E: hematoxylin and eosin; LGE: lateral ganglionic eminence; MCL: mitral cell layer; OB: olfactory bulb; OBLS: olfactory bulb-like structure; OE: olfactory epithelium; OEC: olfactory ensheathing cell; OMP: olfactory marker protein; ONL: outer nerve layer; OR: odorant receptor; OSN: olfactory sensory neuron; PBS: phosphate-buffered saline; PK2: prokineticin 2; vGlut: vesicular glutamate transporter; VZ: ventricular zone.

## Competing interests

The authors declare that they have no competing interests.

## Authors’ contributions

Experiments were conceived and designed by TS and CS, and most were performed by TS with assistance from DD in cell counts. DMK contributed reagents, technical and intellectual assistance. The manuscript was written by TS and CS and edited by all authors. All authors read and approved the final manuscript.
